# Effect of weekend hospitalization on the duration of fasting in patients with aspiration pneumonia

**DOI:** 10.1002/jgf2.564

**Published:** 2022-06-13

**Authors:** Yuki Hasumi, Yoshito Hirota, Masahiro Nishimura

**Affiliations:** ^1^ Department of General Medicine Mogami Municipal Hospital Yamagata Japan; ^2^ Department of Healthcare Economics and Quality Management, Graduate School of Medicine Kyoto University Kyoto Japan; ^3^ Japan Association for Development of Community Medicine (JADECOM) Institute of Community Medicine Chiyoda City Japan

**Keywords:** aspiration pneumonia, fasting duration, weekend hospitalization

## Abstract

**Background:**

Decreased swallowing function is an important risk factor for the development of aspiration pneumonia. A previous study reported that a long duration of fasting decreased swallowing function and increased mortality. The purpose of this study is to clarify the relationship between weekend hospitalization and fasting duration in older patients with aspiration pneumonia.

**Methods:**

In this retrospective cohort study using hospital claims data and electronic medical record data between April 1, 2018, and March 31, 2020, 238 patients hospitalized for aspiration pneumonia were enrolled. Patients admitted from Monday to Friday were defined as the weekday admission group, and patients admitted on Saturday, Sunday, and holidays were defined as the weekend admission group. The primary endpoint was the duration of fasting. Multiple regression analysis was performed with age, gender, place of living, Functional Oral Intake Scale before admission, severity of pneumonia, and weekend admission as the independent variables.

**Results:**

There were 41 patients in the weekend admission group and 103 patients in the weekday admission group. The baseline characteristics of the two groups were similar. There was a median difference in the fasting duration of 23.3 h; the fasting duration in the weekend admission group was significantly longer than the weekday admission group by 20.03 h (95% confidence interval: 2.81–37.25) in the multiple regression analysis.

**Conclusions:**

Weekend hospitalization was significantly associated with a longer fasting duration in patients hospitalized for aspiration pneumonia. There is a need to establish a system to enable early meal initiation for patients admitted on weekends.

## Graphical abstract

We showed that weekend hospitalization was significantly associated with a longer fasting duration in patients hospitalized for aspiration pneumonia. Weekend hospitalization may be an important factor for treating aspiration pneumonia.

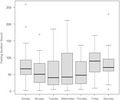

## 1 INTRODUCTION

In 2019, aspiration pneumonia was the sixth leading cause of death in Japan^1^ and is currently a major cause of death in an aging society. In a previous study investigating whether bacterial pneumonia in hospitalized patients was because of aspiration or nonaspiration, aspiration pneumonia occurred in approximately 50% of patients in their 60s, 70% in their 70s, and >80% in their 80s.^2^ As society ages, the number of patients with aspiration pneumonia is expected to increase, and its prevention and treatment are a socially important topic.

Decreased swallowing function is an important risk factor for the development of aspiration pneumonia, and preventing a decline of swallowing function is important for treating aspiration pneumonia.^3^ A previous study found that a long duration of fasting decreased swallowing function, prolonged treatment time, and increased mortality.^4^ To shorten the fasting duration, it is necessary to elucidate the factors that cause a prolonged duration of fasting. However, few studies have examined this issue.

The effect of weekend hospitalization on patient outcomes as compared with that of weekday hospitalization is generally referred to as the “weekend effect.” Weekend hospitalization has been associated with increased mortality because of staff shortage and omissions or delays for appropriate treatment on weekends.^5^ A previous study reported that the weekend effect existed in the management of community‐acquired pneumonia.^6^ However, to the best of our knowledge, no studies have reported the weekend effect in patients with aspiration pneumonia.

We hypothesized that a weekend effect also exists in patients hospitalized for aspiration pneumonia and that weekend hospitalization may cause a long fasting duration, resulting in a decline in swallowing function. The purpose of this study was to clarify the relationship between weekend hospitalization and both fasting duration and swallowing function in older adults hospitalized for aspiration pneumonia at an acute care general hospital.

## 2 METHODS

### 2.1 Study setting and population

We conducted a retrospective cohort study using diagnosis procedure combination (DPC) data and electronic medical record data. Study participants admitted to the Department of General Medicine were enrolled between April 1, 2018, and March 31, 2020, at our hospital, a 343‐bed designated secondary emergency hospital in Tokyo. The inclusion criteria were as follows: (1) patients aged ≥65 years and (2) patients who were recorded as having aspiration pneumonia in the main diagnosis, cause of admission, and the most medical resource‐intensive diagnosis of the DPC data. We excluded the following patients: (1) patients who died during hospitalization, (2) patients who did not eat during hospitalization, (3) patients who were hospitalized for other diseases and misrecorded as having aspiration pneumonia, (4) patients who were admitted to the intensive care unit (ICU)/high care unit (HCU), (5) patients who used ventilators including noninvasive positive pressure ventilation, (6) patients who developed hospital‐acquired pneumonia and were transferred to our hospital, (7) patients who used tube feeding before admission, and (8) patients with missing data in the variables.

In our setting, a hospital general medicine specialist is the attending physician for patients with aspiration pneumonia admitted on weekdays. However, during weekends, physicians on duty manage patients with aspiration pneumonia, and the hospital general medicine specialists take over the duties the following weekday. Speech therapists work during the weekdays but not on weekends. The number of ward nurses on weekends is approximately 20% lesser than weekdays. After the handover, the attending physician or speech therapist conducts a screening evaluation of the patient's swallowing function and determines whether the patient should resume eating.

### 2.2 Measurements

Using the electronic medical record data, we extracted age, gender, date and time of admission, day of admission, date and time of meal initiation, place of living before admission (home or nursing home), and Functional Oral Intake Scale (FOIS) score (before admission and at discharge).^7^ In the FOIS, a higher score indicates that the meal form is closer to that of a normal diet, and a lower score indicates greater difficultly with oral intake and a heavier use of tube feeding. The authors determined the FOIS score by reviewing the meal form that the speech therapist had documented in the electronic medical record. In addition, blood test values (blood urea nitrogen) and vital signs (temperature, saturation of percutaneous oxygen [oxygen dosage], respiratory rate, orientation, and systolic blood pressure) at the time of admission were extracted, and the A‐DROP score was calculated. The A‐DROP score, proposed by the Japanese Respiratory Society based on the British Thoracic Society's CURB‐65 score, is a 6‐point (0–5 scale) scoring system that assesses the severity of community‐acquired pneumonia.^8^ This scoring system assesses the following parameters: (1) age (men ≥70 years, women ≥75 years), (2) dehydration (blood urea nitrogen ≥21 mg/dl), (3) respiratory failure (SaO_2_ ≤ 90% or PaO_2_ ≤ 60 mmHg), (4) orientation disturbance (confusion), and (5) low blood pressure (systolic blood pressure ≤90 mmHg). In addition, we extracted head and neck tumors, history of gastroesophageal surgery, cerebral nerve disease, chronic lower respiratory tract disease, and diabetes from the discharge summary in the electronic medical record data and all the names of diseases recorded in the DPC data, including “the second‐most medical resource–intensive diagnosis,” “comorbidity,” and “complications after admission.”

### 2.3 Outcome measures

The primary endpoint was the duration of fasting from admission to the start of meals, in hours. The secondary endpoint was the change in FOIS between prehospitalization and discharge (the difference between the FOIS at discharge minus the FOIS before hospitalization) to assess the change in meal form because of hospitalization. In this study, we excluded patients who had been on tube feeding before hospitalization, and thus, we included only those with an FOIS score of 4 or higher before hospitalization.

### 2.4 Statistical analysis

Patients were classified into two groups according to the day of admission: weekday admission group and weekend admission group. Patients admitted from Monday to Friday were defined as the weekday admission group, and patients admitted on Saturday, Sunday, and holidays were defined as the weekend admission group. For comparison between the two groups, we performed a *t*‐test or Wilcoxon's rank‐sum test for continuous variables and *χ* ^2^ test for categorical variables as appropriate. Multiple regression analysis was performed with fasting duration and FOIS change as the dependent variables and with age, gender, place of living before admission (home or nursing home for older adults), FOIS before admission, A‐DROP score (0–2 points and 3–5 points), and weekend admission as the independent variables. We selected explanatory variables based on previous studies.^4^, ^9^ We also described the duration of fasting for each day of the week as a boxplot. As a sensitivity analysis, patients admitted after 17:00 on Friday were also classified as the weekend admission group, and multiple regression analysis was performed.

With regard to the sample size, we set a goal of 90 or more patients in reference to previous studies, which reported that 10–15 cases were necessary for one explanatory variable in multiple regression analysis.^10^, ^11^

Statistical analyses were performed using R software version 4.0.2 (R Foundation for Statistical Computing), and a two‐sided significance level of 0.05 was used.

### 2.5 Ethical considerations

The study was approved by the institutional review board of Tokyo‐Kita Medical Center. We did not obtain consent from the patients, because this is a retrospective study. The requirement for consent was waived by the review board. We presented materials about opting out on the hospital's website and provided an opportunity for participants to refuse to participate in the study. We ensured that the anonymity of the participants was preserved. This study was conducted in accordance with the ethical guidelines for medical and health research involving human subjects of the Ministry of Education, Culture, Sports, Science and Technology and Ministry of Health, Labour and Welfare.^12^

## 3 RESULTS

**FIGURE 1:**
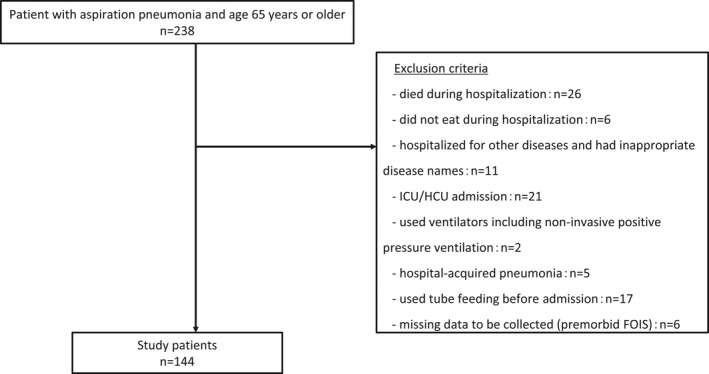
Study flowchart

A total of 238 patients met the inclusion criteria. Of these, 144 patients who did not meet the exclusion criteria were included in the analysis (Figure 1). There were 41 patients in the weekend admission group and 103 patients in the weekday admission group (Table 1). The two groups were similar in terms of age, gender, medical history, place of living before admission, and A‐DROP score. There was no difference in FOIS before admission, FOIS at discharge, or change in FOIS between the groups; however, there was a median difference in fasting duration of 23.3 h.

**TABLE 1:** Patient characteristics and outcomes

	Weekday group (*n* = 103)	Weekend group (*n* = 41)	*p*
Age (years), mean (SD)	86.5 (7.9)	86.2 (8.4)	0.825^a^
Female (%)	52 (50.5)	22 (53.7)	0.874^b^
Comorbidities (%)			
Cerebral nerve disease	88 (85.4)	38 (92.7)	0.364^b^
Chronic lower respiratory disease	26 (25.2)	8 (19.5)	0.608^b^
Diabetes mellitus	25 (24.3)	10 (24.4)	1^b^
Tumor of the head and neck	2 (1.9)	1 (2.4)	1^b^
History of gastroesophageal surgery	6 (5.8)	1 (2.4)	0.672^b^
Premorbid living place (%)			
Home	58 (56.3)	22 (53.7)	0.918^b^
Nursing home for older adults	45 (43.7)	19 (46.3)	
Admission day (%)			
Sunday	0 (0.0)	18 (43.9)	<0.001^b^
Monday	20 (19.4)	2 (4.9)	
Tuesday	24 (23.3)	0 (0.0)	
Wednesday	19 (18.4)	0 (0.0)	
Thursday	20 (19.4)	0 (0.0)	
Friday	20 (19.4)	0 (0.0)	
Saturday	0 (0.0)	21 (51.2)	
A‐DROP score (%)			
≤2	70 (68.0)	29 (70.7)	0.901^b^
≥3	33 (32.0)	12 (29.3)	
Outcomes			
Premorbid FOIS (median [IQR])	5 [4, 6]	6 [5, 6]	0.157^c^
At discharge FOIS (median [IQR])	5 [4, 5]	5 [4, 5]	0.65^c^
FOIS change(median [IQR])	−1 [−2, 0]	−1 [−1, 0]	0.744^c^
Time from admission to intake (h), median [IQR]	47.25 [21.93, 94.29]	70.57 [59.32, 93.28]	0.072^c^

Abbreviations: FOIS, Functional Oral Intake Scale; IQR, interquartile range.

^a^ *p* Value by *t*‐test for continuous variables.

^b^ *p* Value by *χ* ^2^ test for categorical variables.

^c^ *p* Value by Wilcoxon's rank‐sum test for continuous variables.

In the multiple regression analysis for the primary endpoint, the confounding‐adjusted fasting duration in the weekend admission group was significantly longer than that in the weekday admission group by 20.03 h (95% confidence interval: 2.81–37.25 h; Table 2).

**TABLE 2:** Results of the multivariable regression analyses

	Duration of fasting		FOIS change	
	Regression coefficient (95% CI)	*p*	Regression coefficient (95% CI)	*p*
Admission day				
Weekday	Reference		Reference	
Weekend	20.03 (2.81–37.25)	0.024	0.20 (−0.17–0.57)	0.286

*Note:* Regression coefficients were adjusted for age, gender, premorbid living place (home, nursing home), premorbid FOIS, A‐DROP score (≥3, ≤2).

Abbreviations: CI, confidence interval; FOIS, Functional Oral Intake Scale.

In the multiple regression analysis for the secondary endpoints, we found no significant association between FOIS change and weekend hospitalization.

**FIGURE 2:**
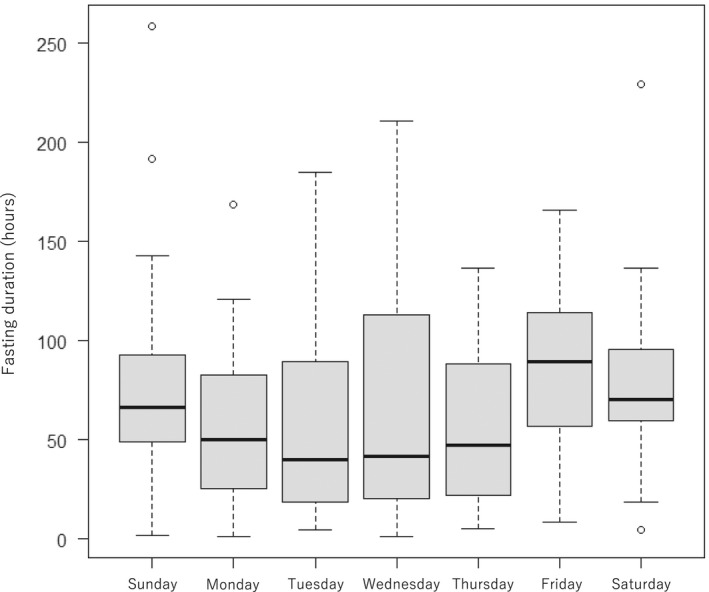
Boxplot of the duration of fasting for each day of the week. Median duration of fasting [interquartile range, IQR] for each day of the week: 66.4 [51.7, 91.7], Sunday; 50.3 [25.9, 79.7], Monday; 39.9 [18.6, 89.1], Tuesday; 41.7 [20.3, 113.0], Wednesday; 47.4 [22.3, 88.6], Thursday; 89.5 [62.4, 113.9], Friday; and 70.6 [59.9, 95.9], Saturday. Abbreviations: ICU, intensive care unit; FOIS, functional Oral intake scale; HCU, high care unit

The median duration of fasting for each day of the week was the longest on Friday and the shortest on Tuesday (Figure 2). In the sensitivity analysis in which patients admitted after 17:00 on Friday were classified as the weekend admission group, the duration of fasting of the weekend admission group was significantly longer than that of the weekday admission group by 22.8 h (95% confidence interval: 6.4–39.2 h) after confounding adjustment (Table 3). However, there was no significant association between change in FOIS and weekend hospitalization in the sensitivity analysis.

**TABLE 3:** Results of the multivariable regression analyses on sensitive analysis

	Duration of fasting		FOIS change	
	Regression coefficient (95% CI)	*p*	Regression coefficient (95% CI)	*p*
Admission day				
Weekday	Reference		Reference	
Weekend	22.83 (6.44–39.22)	0.007	0.01 (−0.35–0.36)	0.960

*Note:* Regression coefficients were adjusted for age, gender, premorbid living place (home, nursing home), premorbid FOIS, A‐DROP (≥3, ≤2).

Abbreviations: CI, confidence interval; FOIS, Functional Oral Intake Scale.

## 4 DISCUSSION

In this study, weekend admission was significantly associated with a longer duration of fasting. It is necessary to examine the factors associated with a longer fasting duration on the weekend. In our setting, the attending physician for patients admitted with aspiration pneumonia on weekdays is a hospital general medicine specialist. However, during the weekends, physicians on duty manage patients with aspiration pneumonia and then hand them over to hospital general medicine specialists on the next weekday. After the handover, the attending physician or a speech therapist conducts a screening evaluation of the patient's swallowing function and determines whether the patient should start eating.

A previous study reported that physicians who manage aspiration pneumonia patients are hesitant to initiate oral intake of patients over the weekend because of concerns about medical resources and how to respond to recurrence.^13^ Similarly, in our setting, we speculate that physicians on duty hesitated to start patient oral intake because there were few physicians and nurses working on weekends, and they were concerned about the medical resources needed to respond to an exacerbation of pneumonia.

The absence of specialists to assess the patient's swallowing function may be the other factor associated with a longer duration of fasting over the weekend. Although the assessment of swallowing function is typically performed by a speech therapist or otolaryngologist, few hospitals have a speech therapist or otolaryngologist on weekends. We speculate that in our setting, physicians on duty hesitated to assess swallowing function of patients with aspiration pneumonia who were hospitalized on weekends and thus postponed the patient's start of oral intake until the next weekday because of the absence of specialists who can perform a swallowing function assessment.

We defined the weekend admission group as patients admitted on Saturdays, Sundays, or holidays. In terms of lack of medical resources, patients admitted after 17:00 on Friday are similar to those hospitalized on weekends. Indeed, the median duration of fasting for each day of the week was the longest on Friday, followed by Saturday and Sunday. Therefore, we performed a sensitivity analysis in which patients admitted after 17:00 on Friday were classified as the weekend admission group. The sensitivity analysis showed similar results to the main analysis, which was the multiple regression analysis for the fasting period. This result suggests that the lack of medical resources on weekends may be associated with a longer fasting duration.

Conversely, although previous studies have shown that a long duration of fasting can cause a decline in swallowing function, our study did not show an association between weekend hospitalization and changes in FOIS. This might be because of a lack of power owing to the small number of cases, the possibility that a difference in fasting duration of approximately 1 day does not lead to changes in the FOIS, or the possibility that the number of assessment categories for the FOIS is small and changes are thus difficult to detect. In addition, the FOIS is only a surrogate index of swallowing function, and the decision regarding meal form may be influenced not only by swallowing function but also by the presence or absence of nursing care, such as meal assistance, living place, and other factors. Furthermore, patients with dysphagia may not follow the instructions of medical personnel for reasons such as not being able to accept their dysphagia, not liking highly viscous foods, or believing that their quality of life will be compromised if they do not eat regular food.^14^ For example, even if medical personnel judge that a paste meal is an appropriate meal form based on swallowing function, patients may consume regular food at their own request, occasionally with aspiration pneumonia. Therefore, because changes in the FOIS may not necessarily correlate with a decline in swallowing function, additional research needs to be conducted after reviewing the indicators to determine whether weekend hospitalization causes a decline in swallowing function.

Although in this study, we did not find a significant association between weekend hospitalization and decreased swallowing function, a previous study^9^ reported that the weekend meal initiation group had better outcomes in terms of an improvement in the rate of discontinuation of oral intake because of aspiration after meal initiation and a shorter duration of hospitalization as compared with the weekday meal initiation group. Establishing a system that allows for early meal initiation over the weekend in patients hospitalized with aspiration pneumonia may be an effective intervention to prevent prolonged fasting, a resulting decline in swallowing function, prolonged hospitalization, and increased mortality. However, a simple assessment of swallowing function has not yet been established to help nonspecialists in the field of dysphagia determine the form and timing of food initiation.^15^ Several scoring systems have been tested extensively, such as the Gugging Swallowing Screen and a two‐step thickened water test^16^, ^17^; however, these systems are somewhat cumbersome to use for simple screening when there are few personnel available. To enable the earlier initiation of eating, a flowchart is required as an initial screening that can be easily implemented in approximately 5–10 min even by nonprofessional medical personnel and that can determine the meal form.

## 5 LIMITATIONS

This study was conducted at a single institution, and there are limitations to its generalizability to other institutions. To extract patients with aspiration pneumonia, we used cases in which aspiration pneumonia was entered in the DPC data as the main diagnosis, cause of admission, and most medical resource‐intensive diagnosis. It is possible that cases of aspiration pneumonia in which sepsis or respiratory failure were entered in any of the three disease names were omitted. However, this study excluded patients admitted to the ICU or HCU and those on ventilators, including noninvasive positive pressure ventilation. Patients with an input of sepsis or respiratory failure might be included in the exclusion criteria, so the results are not expected to change significantly.

## 6 CONCLUSIONS

Weekend hospitalization was significantly associated with a longer duration of fasting in patients hospitalized for aspiration pneumonia. There is a need to establish a system to enable early meal initiation for patients admitted on weekends.

## CONFLICT OF INTEREST

The authors have stated explicitly that there are no conflicts of interest in connection with this article.
